# Forest floor community metatranscriptomes identify fungal and bacterial responses to N deposition in two maple forests

**DOI:** 10.3389/fmicb.2015.00337

**Published:** 2015-04-23

**Authors:** Cedar N. Hesse, Rebecca C. Mueller, Momchilo Vuyisich, La Verne Gallegos-Graves, Cheryl D. Gleasner, Donald R. Zak, Cheryl R. Kuske

**Affiliations:** ^1^Bioscience Division, Los Alamos National LaboratoryLos Alamos, NM, USA; ^2^Department of Ecology and Evolutionary Biology, School of Natural Resources and Environment, University of MichiganAnn Arbor, MI, USA

**Keywords:** metatranscriptomics, nitrogen deposition, soil fungal community, soil bacterial community, carbohydrate active enzymes, soil RNA

## Abstract

Anthropogenic N deposition alters patterns of C and N cycling in temperate forests, where forest floor litter decomposition is a key process mediated by a diverse community of bacteria and fungi. To track forest floor decomposer activity we generated metatranscriptomes that simultaneously surveyed the actively expressed bacterial and eukaryote genes in the forest floor, to compare the impact of N deposition on the decomposers in two natural maple forests in Michigan, USA, where replicate field plots had been amended with N for 16 years. Site and N amendment responses were compared using about 74,000 carbohydrate active enzyme transcript sequences (CAZymes) in each metatranscriptome. Parallel ribosomal RNA (rRNA) surveys of bacterial and fungal biomass and taxonomic composition showed no significant differences in either biomass or OTU richness between the two sites or in response to N. Site and N amendment were not significant variables defining bacterial taxonomic composition, but they were significant for fungal community composition, explaining 17 and 14% of the variability, respectively. The relative abundance of expressed bacterial and fungal CAZymes changed significantly with N amendment in one of the forests, and N-response trends were also identified in the second forest. Although the two ambient forests were similar in community biomass, taxonomic structure and active CAZyme profile, the shifts in active CAZyme profiles in response to N-amendment differed between the sites. One site responded with an over-expression of bacterial CAZymes, and the other site responded with an over-expression of both fungal and different bacterial CAZymes. Both sites showed reduced representation of fungal lignocellulose degrading enzymes in N-amendment plots. The metatranscriptome approach provided a holistic assessment of eukaryote and bacterial gene expression and is applicable to other systems where eukaryotes and bacteria interact.

## Introduction

Atmospheric deposition of nitrogen (N) from anthropogenic sources has been increasing and this trend is predicted to continue in temperate regions of Earth (Galloway et al., 2004; Tørseth et al., 2012). The ability of N-limited forest soils to operate as a carbon sink under increased N deposition, through increased incorporation of plant litter and belowground plant biomass, has been suggested as a means to offset predicted rises in atmospheric trace gasses, thus ameliorating the magnitude of climate warming (Reay et al., 2008; Liu and Greaver, 2010). However, the responses of temperate forests to anthropogenic N deposition have been mixed. Although most studies show an increase in plant growth (net primary productivity) under N deposition, soil C storage has not always increased and the microbial dynamics mediating litter decomposition and soil C cycling under ambient or N deposition conditions remain unclear (Janssens et al., 2010; Liu and Greaver, 2010).

Decomposition of plant litter deposited to the soil floor is a central step in C cycling for all terrestrial ecosystems. It is especially important in perennial ecosystems such as temperate forests, in which large amounts of plant litter accumulate on the forest floor and enrich the upper soil horizons with C and N. This process is conducted by a hyper-diverse group of fungi, other eukaryotes, bacteria, and archaea that are highly interactive, and that have potential to respond rapidly and dramatically to changes in environmental conditions such as atmospheric N deposition (reviewed by Osono, 2007; Prescott and Grayston, 2013, examples specific to our study sites in Eisenlord and Zak, 2010; Edwards et al., 2011). Fungi and bacteria secrete extracellular enzymes that enable decomposition of recalcitrant carbon compounds such as cellulose, lignin, and chitin and play major roles in litter decomposition (Osono, 2007; Baldrian, 2014). Enzymes from different fungal and bacterial species have different affinities for plant biomass substrates; their ability to act on different substrates, the rates by which they are able to decompose different substrates, and the participation in different stages of decomposition differ significantly among species of bacteria and fungi. Secreted enzymes from one species may make down-stream substrates available to multiple other organisms. Thus, the decomposition process is achieved through the collective activities of the fungal and bacterial communities and must be treated holistically.

The relative contributions and importance of forest floor fungi and bacteria to *in situ* decomposition are unknown, and understanding their respective activities in C cycling is critical toward calculation of C budgets contributed by temperate forests. Currently C models differ significantly in predicting carbon storage or increased release from soils in terrestrial ecosystems under altered climate scenarios (Friedlingstein et al., 2014), in part because the magnitude and the direction of the soil C and N cycling processes to altered N deposition or other climate variables remains unknown and has been difficult to predict based on current information.

Previous studies of the impacts of N deposition on decomposition have targeted specific decomposer populations or enzymes, namely the Actinobacteria (e.g., Eisenlord and Zak, 2010; Eisenlord et al., 2013; Freedman and Zak, 2014), certain fungal groups and specific fungal enzymes (Waldrop et al., 2004; Zak et al., 2008; Hassett et al., 2009; Edwards et al., 2011; Entwistle et al., 2013). Although interesting shifts and trends have been noted in components of the forest floor community in response to N deposition, it has been difficult to interpret the broader ecosystem consequences of N deposition on decomposition and other surface soil processes using targeted studies only. It has not been possible to interpret these responses in the context of the entire community so the realistic magnitude of response remains unknown. Holistic surveys of the community through metatranscriptomes that simultaneously survey the activities of all microbiota may be better able to place the activities of individual members of the community in context and provide a more accurate assessment of the *in situ* processes (Kuske et al., 2015).

The ability of transcriptomic approaches in environmental samples to survey metabolic genes has been hindered by methodological considerations. Total RNA extracted from environmental samples is highly enriched in ribosomal RNA (rRNA) (Urich et al., 2008; Tveit et al., 2013) that must be removed to efficiently sequence the expressed gene pool (mRNA). This can be accomplished for the Eukarya using polyA enrichment approaches that select for the eukaryotic poly-adenylated (polyA) mRNA in a total RNA sample (Bailly et al., 2007; Weber and Kuske, 2011; Damon et al., 2012). However, a comprehensive assessment of the decomposer community requires inclusion of the non-adenylated bacterial mRNA in addition to the polyA eukaryote mRNA, and these would be missed in surveys using a polyA enrichment approach.

The objectives in this study were three-fold. First, we used a community metatranscriptome survey approach based on Illumina sequencing that substantially reduced rRNA content and provided a high-coverage profile of community transcribed genes with good representation of both the non-polyA and poly-A mRNA from highly decomposed forest floor substrates. Second, we validated this approach using a suite of read-based assessments to document rRNA removal, determine domain identity mRNA sequences using UniProt, and metabolic coverage of the community transcriptomes using KEGG mapping. Third, we extracted the carbohydrate active enzymes (CAZymes) from the metatransriptomes and conducted a comparison of the maple forest floor communities in two natural forests and shifts in CAZyme profiles after 16 years of experimental N deposition. We focused this study on bacterial and fungal communities, as these groups are responsible for a large proportion of ecosystem functions in terrestrial ecosystems (van der Heijden et al., 2008).

Chronic experimental N deposition has been studied for almost two decades in four sugar maple-dominated forests of the Great Lakes region of North America (Zak et al., 2008). Over the years, the forests have responded to N deposition with slightly increased net primary productivity, but no increase in litter production (Burton et al., 2004; Pregitzer et al., 2008b). The same forests exhibited reduced litter decay, increased soil organic matter, and accelerated leaching of dissolved organic carbon (Pregitzer et al., 2004, 2008a), suggesting a reduced ability of the forest floor microbial community to decompose plant litter under N deposition. Our hypotheses for the CAZyme comparison were (a) the complex community in the forest floor material would show responses to N deposition that were manifest in both the bacterial and fungal CAZyme activity profiles, and (b) that the metatransriptomes would illustrate community metabolic shifts that were not necessarily detectable in broader taxonomic surveys based on the bacterial or fungal rRNA.

## Materials and methods

### Site description and sample collection

The impact of chronic atmospheric N deposition on four natural hardwood forest stands located in Michigan, USA has been studied for many years in a long-term experiment. Extensive information regarding geographic location, ambient N deposition, floristic composition, and soil conditions has been published previously (Pregitzer et al., 1992, 2008b; Zak et al., 2011; Eisenlord et al., 2013; Talhelm et al., 2013). Two of the sites were used in the current study. Site Pellston (N45°33′, W84°51′, termed site B in the prior studies, e.g., Eisenlord et al., 2013; Talhelm et al., 2013) is located 145 km north of site Wexford (N44°23′, W85°50′, termed site C in the prior studies; e.g., Eisenlord et al., 2013; Talhelm et al., 2013). When the N deposition experiments began, natural forest stands were matched as much as possible for age (91–92 yr), overstory biomass, and edaphic factors. Both stands are dominated by sugar maple (*Acer saccharum* Marsh). The north Pellston site also contains *Fraxinus americana* L., and the south Wexford site contains *Fagus grandifolia* Ehrh., *Prunus serotina* Ehrh., and *Quercus rubra* L. (Pregitzer et al., 1992; Talhelm et al., 2013). Differences in groundcover, understory plants and edaphic properties measured beneath the forest floor horizon also characterize the two sites, with higher soil calcium, pH (5 vs. 4.5), cation exchange capacity (3.8 vs. 2.6 mmol c/kg) and base saturation (96 vs. 73%) at Pellston compared to Wexford (Eisenlord and Zak, 2010; Talhelm et al., 2013).

Six 30-m by 30-m plots are established at each of the two forest sites, with three plots at each site receiving ambient N deposition (Pregitzer et al., 1992), and three plots receiving an additional 3 g NO_3_-N m^2^ year^−1^ applied twice over the growing season, once in May and once in September. The treatments were initiated in 1994 and have been maintained to the present. Ten random replicate forest floor samples were collected by hand from a 10 cm by 10 cm frame within each forest field plot in October 2011. The intermediately to moderately decomposed (Oe/Oa) horizon, representing prior years' leaf fall, was collected down to the mineral soil as described by Eisenlord et al. (2013). The 10 field replicate samples were pooled immediately after collection and homogenized with sterilized scissors to generate a composite “plot” sample, providing a total of 12 field plot samples in the two forest sites (three ambient and nitrogen plots in the two forests). A 10 g subsample was frozen in liquid N_2_ prior to DNA and RNA extraction. The collected material contained highly decomposed broadleaf leaves with some leaf structure still visible.

### Nucleic acid extraction, RNA purification and testing for RNA purity

For each of the 12 composite field samples, total DNA and RNA were co-extracted from three 2 g subsamples and then pooled after extraction. Each subsample was ground in liquid N_2_ to a fine powder, and total DNA and RNA were co-extracted using a modified bead mill homogenization and organic extraction method based on Griffiths et al. (2000). Ground samples were placed in 15 ml bead tubes (Mo-Bio RNA PowerSoil Total RNA isolation kit, MoBio, Carlsbad CA, USA) with 2.0 ml CTAB extraction buffer (10% CTAB in 0.7 M NaCl 1:1 with 240 mM potassium phosphate buffer, pH 8.0) and 2.5 ml of phenol:cholorform:isoamyl alcohol (25:24:1, pH 8.0). The mixture was shaken using a vortex adapter for 3 min at maximum speed and centrifuged at 2500 × g for 10 min. The upper aqueous phase was extracted with phenol:isoamyl alchohol (24:1, pH 8) and the sample was centrifuged at 2500 × g for 5 min. Nucleic acids in the upper aqueous phase were precipitated with sodium acetate and isopropanol and suspended in RNase-free deionized water. The three subsamples were combined into a single tube, and then partitioned into equal volume aliquots for RNA and DNA isolation.

DNA was removed from half of the sample using Turbo DNase I enzymatic digestion (Ambion Life Techonologies, Carlsbad CA, USA). The resulting RNA was purified using the RNeasy purification kit (Qiagen, Venlo, Netherlands), and eluted in RNase-free water. To ensure complete removal of DNA, samples were treated with the RNase-free DNase set (Qiagen), cleaned using the RNeasy purification kit (Qiagen) and eluted into RNase-free water. RNA concentration was determined using a Nanodrop spectrophotometer (ThermoScientific, Waltham MA, USA). The purified RNA was again subdivided into two aliquots, one for qPCR and one for cDNA metatranscriptome and ribosomal amplicon sequencing.

Prior to metatranscriptome sequencing, a quantitative real-time PCR (qPCR)-based assay was used to determine if residual DNA was present in the purified RNA preparations. For each RNA sample, two individual reactions were conducted: one containing reverse transcriptase (+RT) and one without reverse transcriptase (-RT). RNA samples were normalized to a concentration of 0.1 ng/μl and a single master mix containing SYBR Green and DNA polymerase was used for all reactions. The 16S ribosomal RNA gene (rRNA gene) was targeted with primers Eub338F and Eub514R (Amann et al., 1995) in the +RT and –RT reactions. The +RT PCR reaction contained 12.5 μl iScript One-step RT PCR master mix with SYBR Green (BioRad, CA, USA), 0.8 μl 5 μM of each primer, 0.3 μl Bovine Serum Albumin (20 mg/mL, Roche Applied Science, IN, USA), 0.5 ul reverse transcriptase, 9.1 ul deionized water, and 1 ul RNA extraction solution. The –RT PCR reaction solution was identical to the +RT reaction, with the omission of 0.5 ul reverse transcriptase. Both samples were cycled on a BioRad CFX thermocycler while monitoring SYBR green fluorescence using the following cycling program: Initial denaturing steps of 50°C for 10 min followed by 95°C for 5 min; followed by 40 cycles of (a) 94°C for 15 s to denature the templates, (b) 55°C for 30 s to anneal primers, and (c) 72°C for 30 s for extension. The observation of a 10 cycle or greater difference in amplification efficiency between +RT and –RT reactions was used as a cutoff for sufficient DNA removal in RNA samples.

### Taxonomic surveys using the ribosomal RNA gene (rDNA) and ribosomal RNA (rRNA)

#### Quantitative PCR of fungal 18S rRNA and bacterial 16S rRNA

Quantitative PCR was used as a relative measure of fungal or bacterial biomass across the experimental field plots. Prior to quantitative PCR (qPCR), all samples (RNA and DNA) were normalized to 0.1 ng μl^−1^ concentration. For the qPCR reactions with RNA template, complementary DNA (cDNA) was generated using the iScript One-Step RT PCR Kit (BioRad). A control reaction, where the reverse transcriptase enzyme (RT) was omitted (termed the no-RT control), was conducted in parallel for each RNA template to ensure that amplification was not from contaminating DNA. Fungal and bacterial qPCR was performed on a CFX Connect Real-Time PCR Detection System (BioRad Laboratories, Hercules, CA) with the reaction mix and conditions described previously (Steven et al., 2013). The fungal quantitative standard was a cloned Agaricales 18S rRNA gene sequence, and the bacterial standard was a cloned Mycobacterium 16S rRNA gene sequence.

#### Sequencing fungal and bacterial ribosomal RNA genes (rDNA) from forest floor material

rRNA genes were prepared for 454 pyrosequencing by PCR amplification with 8 bp MID tags inserted into the reverse primer. The D1-D2 hyper-variable region of the fungal LSU rRNA gene was targeted with the primer pair LR0R-LR3 (Vilgalys and Hester, 1990), and bacteria were targeted by using the V5-V6 region of the bacterial 16S rRNA gene with the primer pair F784-R1064 (Claesson et al., 2010). PCRs were performed in triplicate 50 μl reactions with thermocycling conditions described by Steven et al. (2013), pooled by field sample and quantified using the PicoGreen kit (Invitrogen, Carlsbad CA, USA). Fungal and bacterial DNA libraries were each sequenced separately on one-half of a sequencing run using 454 FLX Titanium technology (Roche).

Sequences were processed using PyroNoise (Quince et al., 2009) and quality filtered to remove any sequence that was less than 200 bp in length, had an average quality score below 25, or any ambiguous base using the program mothur (Schloss et al., 2009). Downstream sequence processing was conducted using the program QIIME (Caporaso et al., 2010). DNA libraries generated from fungi or bacteria were de-multiplexed separately and subsequently combined for clustering with USEARCH (Edgar, 2010) to remove putative chimeras, singleton sequences, or any sequence with a mismatch to the reverse primer sequence (LR3 or R1064 for fungi and bacteria, respectively). Operational taxonomic units (OTUs) were delineated at 97% sequence similarity using UCLUST (Edgar, 2010), and representative bacterial 16S and fungal LSU OTUs were classified against their respective databases using the online classification tool from the Ribosomal Database Project (Cole et al., 2009; Liu et al., 2012). Any OTU classified as non-Fungi or non-Bacteria was removed from downstream analysis. Raw environmental sequences generated for this study can be accessed in the MG-RAST database (Meyer et al., 2008) under 4557518.3 (Fungal LSU) and 4557516.6 (Bacterial 16S).

#### Statistical analysis of rDNA data

Community and statistical analyses were conducted in the statistical platform R (R Development Core Team, 2014; r-project.org). Two of the bacterial samples had poor sequencing success (<300 sequences) and were removed. To reduce the effects of unequal sequencing depth, samples were rarefied to the minimum number of sequences recovered in a single sample, 900 sequences for fungi and 400 sequences for bacteria, for all statistical analyses. To reduce the effects of incomplete sampling, each analysis was conducted based on values generated from 99 separate rarefactions. Extracted DNA, extracted RNA, qPCR, and RT-qPCR data were analyzed using a factorial ANOVA (model = site, N-amendment, site ^*^ N-amendment), followed by *post hoc* pairwise *t*-tests. *T*-test *p*-values less than 0.05 were considered significant. Fungal OTU composition (97% sequence similarity) was visualized by non-metric multidimentional scaling (NMS) ordination generated using Bray-Curtis dissimilarity. Community composition based on OTUs was compared using PERMANOVA (Anderson, 2001) using the vegan library (Oksanen et al., 2007).

### Metatranscriptome sequencing and analysis

#### rRNA removal and cDNA sequencing

rRNA was removed from the total RNA via hybridization to biotinylated probes provided in Ribo-Zero kits (EpiCentre, WI, USA). Metabacteria and eukaryote (human/mouse/rat) probes were combined, hybridized to total RNA, and the complexes were removed with the magnetic beads provided in the Ribo-Zero kits. Samples were processed according to the manufacturer's protocols with the following modifications: A maximum of 2.5 μg total RNA was used as starting material, 5 μl of rRNA Removal Solution was used from each kit (metabacteria and human/mouse/rat), and final Ribo-Zero purification was accomplished using the RNeasy protocol outlined in the Ribo-Zero manual, eluting in 14 μl RNase-free water. RNA samples were quantified using a Life Technologies RNA quantification kit and a Qubit 2.0 instrument (Life Technologies). Size distribution of the RNA molecules in each sample was determined using a BioAnalyzer 2100 (Agilent) and RNA pico chips and reagents (Agilent). A maximum of 50 ng of RNA from each sample was converted to a Illumina sequencing library using ScriptSeq kits (EpiCentre). Each library was generated using a unique 6 bp multiplex barcode provided in the ScriptSeq kit. Following library generation, the 12 samples, plus two control samples where rRNA had not been removed, were pooled in equimolar concentrations and run on two lanes of the Illumina HiSeq2000 sequencing platform. Paired-end 101 bp reads were obtained.

#### Metatranscriptome data quality and cDNA read annotation control

Base-calls and quality scores were processed using the standard Illumina software and binned according to multiplex barcode prefix. Sequences were trimmed to remove low quality base-calls (*Q* < 30) and any bases following a low-quality base (3-prime trimming). Additionally, reads with internal or tailing uncalled bases (“N”) were removed (FastX Toolkit software, http://hannonlab.cshl.edu/fastx_toolkit). Sequences of at least 85 bp were retained for analyses. Sequences containing portions of the rRNA operon were identified using SortMeRNA and removed from the datasets (Kopylova et al., 2012). Reference databases provided with SortMeRNA from RFAM (Griffiths-Jones et al., 2003; Gardner et al., 2009) (5S and 5.8S ribosomal subunits, 98% identity clustering) and SILVA (Pruesse et al., 2007; Quast et al., 2013) (16S/18S, 95% clustering for archaeal 16S and eukaryotic 18S, 85% clustering for bacterial 16S; 23S/28S, 98% clustering) were used for homology searches. Quality-filtered mRNA sequences are available from the MG-RAST webserver under accessions 4539662.3 through 4539673.3.

Our attempts at *de novo* assembly of these metatranscriptome reads yielded only contigs of very short length (125–175 bp; data not shown) and of questionable quality, therefore we opted for a read-based analysis that was facilitated by the BLASTX algorithm implemented in the USEARCH package (version 6, licensed 64-bit) and condensed protein databases for KEGG (via HUMANN software team), UniProt (UniRef50), and CAZy (dbCAN). As a validation of our approach to simultaneously generate metatransriptomes containing bacterial, fungal and archaeal transcripts, we first conducted cDNA read identification with UniRef50 to determine the representation of bacterial, fungal, and archaeal transcripts in the datasets. We followed with mapping reads to KEGG orthology (KO) to determine how comprehensive the metatransripts were with respect to known bacterial and fungal metabolism. Reduced complexity databases were used where available to minimize computational burden. Read matches with a global alignment *e*-value less than or equal to 1e-5 were retained as putative read annotations. Taxonomic affinities of reads were assigned using the least common ancestor (LCA) algorithm as implemented in MEGAN (Version 5.1.0 build 14, Huson et al., 2007) with the following parameters: min score = 40, min support = 5. KO numbers were obtained using the MEGAN software package and a custom generated map file for the reduced KEGG database. Paired-end sequences were left un-joined throughout the analysis, as the homology searching algorithms used are not optimized for paired-end data.

##### Protein identification using UniRef50

Reads were queried against the UniRef50 protein database (Suzek et al., 2007) using UBLAST with an *e*-value threshold of less than or equal to 1e-5. Results were imported into MEGAN for LCA assignment along with a UniRef50 to NCBI taxonomy mapping file. The taxonomy tree was collapsed to “super kingdom” and read assignments were exported in tabular format for eukaryote-to-bacteria ratio calculation.

##### KEGG analysis

Quality-filtered data were queried against a reduced KEGG database (Kanehisa and Goto, 2000; Kanehisa et al., 2014; Release 56, 2011 with proteins clustered at similarity threshold 85%) provided by the maintainers of the HUMANN analysis package (Abubucker et al., 2012). The translated UBLAST search algorithm from the USEARCH package was used for homology comparison of quality-trimmed reads (nucleotide) to the reduced KEGG database (amino acid). Matches with *e*-values less than or equal to 1e-5 were retained as putative identifications. UBLAST results were imported into the MEGAN software package along with a custom generated map file for the reduced KEGG database to KO numbers. Read matches were inspected and a sample by KO abundance matrix subsequently exported from MEGAN for further analysis. KEGG pathways were visualized using the KEGG database website (http://www.kegg.jp) and the KO abundance matrix for the sum of all samples.

##### Carbohydrate Active Enzymes (CAZyme) analysis

Our main objectives were to identify the contributions of bacterial and fungal metabolism to forest floor decomposition processes, and to determine if these had shifted in response to long-term N-amendment conditions. We therefore focused our analysis of community transcripts on the CAZymes that are involved in decomposition of plant and fungal substrates. A database of full-length CAZymes (Cantarel et al., 2009; Lombard et al., 2014) was obtained from the dbCAN database (Yin et al., 2012). This database represents all proteins from the NCBI Genbank servers that have been identified as CAZymes by the CAZy project (http://www.cazy.org). UBLAST was used to query quality-filtered reads against the dbCAN amino acid database, retaining only matches with *e*-values less than or equal to 1e-5. Summing significant hits to CAZyme families across all reads in a sample generated a matrix of putative CAZyme abundances. Many proteins can contain multiple CAZyme signature domains (i.e., CBM1, GH#); thus a single read could be attributed to multiple CAZyme terms. The concatenation of individual sample tallies resulted in a final matrix that represented 311 CAZyme families and 12 unique samples.

CAZyme associated reads were taxonomically assigned in MEGAN and the supplied NR-database-to-NCBI-taxonomy file was used for LCA taxonomy assignment. The taxonomy tree was collapsed to “super kingdom” and read assignments were exported in tabular format for further analysis. Similarly, taxonomic assignments for select CAZyme genes of interest were exported at phylum level within the Fungi.

#### Statistical analysis of CAZYmes in the metatransriptomes

The CAZyme abundance matrix was normalized to the number of reads per one million annotated mRNA to account for differences in sequencing depth among samples. The NMDS ordination was generated in the R software package (R Development Core Team, 2014) using the vegan library (Oksanen et al., 2007) with the *metaMDS* function and Bray-Curtis dissimilarity metric on square-root transformed data with 1000 iterations for a two-dimensional solution (final stress = 0.10). CAZyme correlations with grouped samples were calculated with the *envfit* function using 1000 permutations. Sample clustering was analyzed for significance using PERMANOVA with 1000 permutations. A heat map of CAZyme family abundances per sample was generated with the ggplot2 library in the R analysis package using abundance data transformed to show the relative difference from the CAZyme family mean. CAZyme abundance correlations were generated using the *cor* function in R with the Pearson correlation method on Wisconsin double standardized abundance data.

## Results

### Sequence quality and dataset description

The subtractive hybridization technique used in this study to deplete rRNA from the total RNA sample was an essential step in obtaining sufficient mRNA sequencing depth for comparison. An average of 17 M total, high quality sequence reads per sample were obtained (treatment averages in Table 1). This corresponded to approximately 56% of the total sequences generated. The combination of bacterial and eukaryotic RiboZero probes reduced the rRNA sequences from an average of 95% in forest floor samples that were sequenced without the RiboZero treatment (data not shown), to an average of 38% in RiboZero treated samples (Table 1).

**Table 1:** **Averaged sequencing, rRNA removal, and annotation statistics**.

**Site**	**Treatment**	**Average reads after QC (SD)**	**Average rRNA %**	**Average mRNA %**	**Average CAZy of mRNA^a^%**	**Eukaryote: bacteria CAZy^b^**	**Average KEGG of mRNA^c^%**	**Average UniRef50 %**	**Eukaryote: bacteria UniRef50^d^**
Pellston	Ambient	15,213,485 (5,159,659)	51	49	0.79	3.39:1	7.80	9.26	3.25:1
Pellston	Nitrogen	19,207,172 (3,301,594)	31	69	0.73	2.35:1	8.17	9.81	3.15:1
Wexford	Ambient	15,093,835 (7,955,069)	38	62	0.67	2.94:1	5.63	7.18	2.30:1
Wexford	Nitrogen	18,518,909 (153,181)	33	67	0.62	2.16:1	5.99	7.32	1.92:1
Average		17,008,350 (5,596,249)	38	62	0.71	2.71:1	6.89	7.03	2.6:1

a Different by Site (*p* = 0.034, 2-sided t-test after ANOVA);

b Different by Treatment at the Pellston site (*p* = 0.041, 2-sided t-test following ANOVA);

c Different by Site (*p* = 3.68e-5, 2-sided t-test after ANOVA;

d *Different by Site (*p* = 2.47e-5, 2-sided t-test after ANOVA)*.

### Gene annotation and taxonomic distribution

As a validation of our metatranscriptome approach we annotated mRNA reads to multiple gene databases. An average of 10.6 M mRNA sequence reads were obtained per sample (~62% of the total high quality reads; Table 1). On average, 7% of the nearly 130 M mRNA sequence reads had a significant match to a cluster in the UniRef50 protein database. Similar proportions of reads had matches to a database of KEGG motif-containing proteins (Table 1). Across all samples, 0.71% of the reads could be attributed to CAZyme families (about 74, 000 reads per sample). There was a consistent site effect for the proportion of reads that could be annotated. Pellston samples had, on average, higher percentages of reads that could be assigned to CAZyme, KEGG, or UniRef50 families than Wexford samples (*t*-test *p* < 0.05 for all comparisons, Table 1). Using the LCA algorithm implemented in MEGAN, we calculated eukaryote to bacteria ratios for CAZyme and UniRef50 genes. Both eukaryote and bacterial genes were well represented in the metatranscriptome datasets. On average, there were more than twice as many CAZyme and UniRef50 reads attributed to eukaryotes than to bacteria (Table 1). The eukaryote to bacteria ratio for CAZyme reads at Pellston was significantly higher than at Wexford (*p* = 0.041, two-sided *t*-test). Of the UniRef50-identified genes, only 0.2% represented either archaea or viruses, and we did not continue analysis of these reads.

### KEGG pathways

To explore the metabolic range of annotated reads in the metatranscriptomes, we mapped read abundances to the metabolic pathways from the KEGG project. Nearly 9 M sequence reads from the 12 samples could be associated with a KEGG enzyme using our sequence homology approach. KEGG metabolic pathways populated with metatranscriptome data show nearly complete pathways for major core metabolic pathways (Supplementary Image 1; file 122812_Kuske_Image 1.png; legend in file 122_Kuske_Presentation 3.pdf), suggesting that the sequencing depth was sufficient to include a breadth of basic metabolic activities. The most highly represented and most complete pathways included those for energy metabolism, carbohydrate metabolism, amino acid metabolism, and nucleotide metabolism. Peripheral pathways were less complete including those for glycan biosynthesis and metabolism, xenobiotics biodegradation, and biosynthesis of other secondary metabolites.

### CAZyme expression profiles

The UniRef50 and KEGG annotations provided validation that our metatranscriptome approach simultaneously detected a broad range of basic metabolic functions from both bacteria and eukaryotes. We focused our remaining analyses on the enzymes associated with plant and fungal biomass degradation using the highly curated CAZy database (Lombard et al., 2014), to explore the relative contributions of bacteria and fungi to forest floor decomposition and to determine if N-amendment had caused shifts in community metabolism. An average of 74,030 sequences per sample could be mapped to a CAZyme family, a sequencing depth that permitted statistically valid sample comparisons.

**Figure 1:**
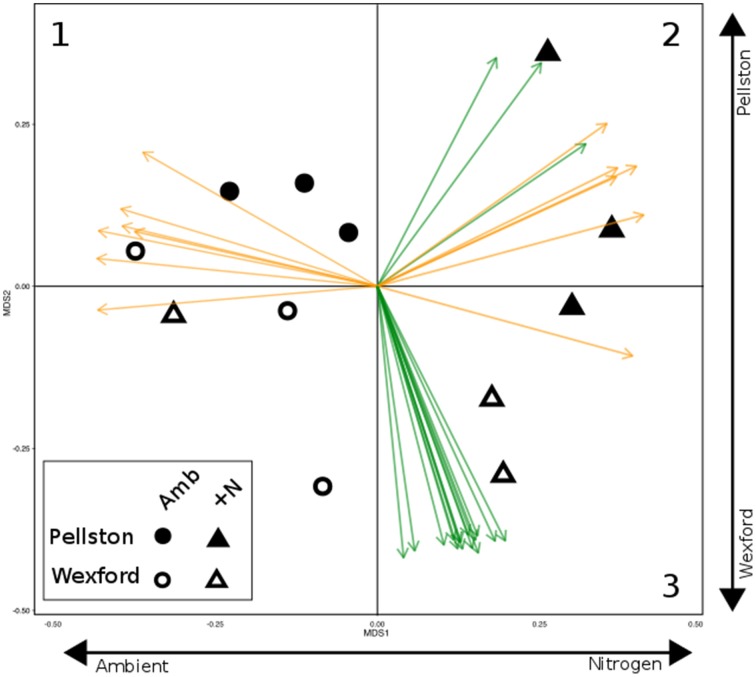
**Non-metric multidimensional scaling ordination of field sample metatranscriptomes based on CAZyme transcript composition**. Samples ordinated first by N treatment (horizontal axis) and second by site (vertical axis). Vectors denote highly significant correlation with ordination axes (*p* < 0.001). Orange vectors represent eukaryotic CAZymes and green vectors represent bacterial CAZymes. Numbers in the three quadrants illustrate CAZymes that are noted in the Figure 2 heat map. [1, eukaryote CAZymes enriched in ambient plots in both forests; 2, eukaryotic and bacterial CAZymes enriched in Pellston N-amended plots; 3, bacterial CAZymes enriched in Wexford N-amended plots]. Clustering of ambient and N-amended plots was significantly different from one another (PERMANOVA, N amendment *p* = 0.02) with a significant interaction between site and treatment (*p* = 0.039). *Post-hoc* tests revealed the effect of treatment was highly significant at Pellston (*p* = 0.001) and not significant at Wexford (*p* > 0.1). See Supplementary Table S1 for names and functions of individual vectors.

The NMS ordination of samples derived from the total set of CAZyme family abundances revealed shifts in expressed CAZyme patterns with site and N amendment (Figure 1). The N deposition treatments partitioned along the horizontal axis, and a non-overlapping separation of samples divided the two forest locations from one another along the vertical axis. PERMANOVA analysis revealed a statistically significant effect of nitrogen amendment (*p* = 0.02) and site^*^nitrogen interaction (*p* = 0.039) but non-significant effect of site on CAZyme expression profiles. *Post-hoc* tests showed N amendment was highly significant at Pellston (*p* = 0.001) and not significant at Wexford (*p* > 0.1). Although 203 (of 453) CAZyme families were significantly correlated with the two ordination axes, joint plot vectors are displayed only for highly significant (*p* < 0.001) correlations of CAZyme family abundances with ordination axes (Figure 1). Seven eukaryote CAZyme families (Figure 1, orange vectors in quadrant 1; GH6, GH7, PL1, AA9, CBM1, CE1, and GH5) were significantly associated with the horizontal axis, indicating their higher abundance in most ambient samples relative to the experimental N deposition treatment. At the northern field site (Pellston), nine CAZyme families that include both bacteria (green vectors) and eukaryotes (orange vectors) were highly correlated with the experimental N deposition treatment (Figure 1, quadrant 2; Supplemental Table S1 for family name and functions). In contrast, at Wexford to the south, a suite of 15 bacterial CAZyme families was highly associated with the experimental N deposition treatment, with no fungal CAZyme families represented (Figure 1, green vectors in quadrant 3; Supplemental Table S1).

**Figure 2:**
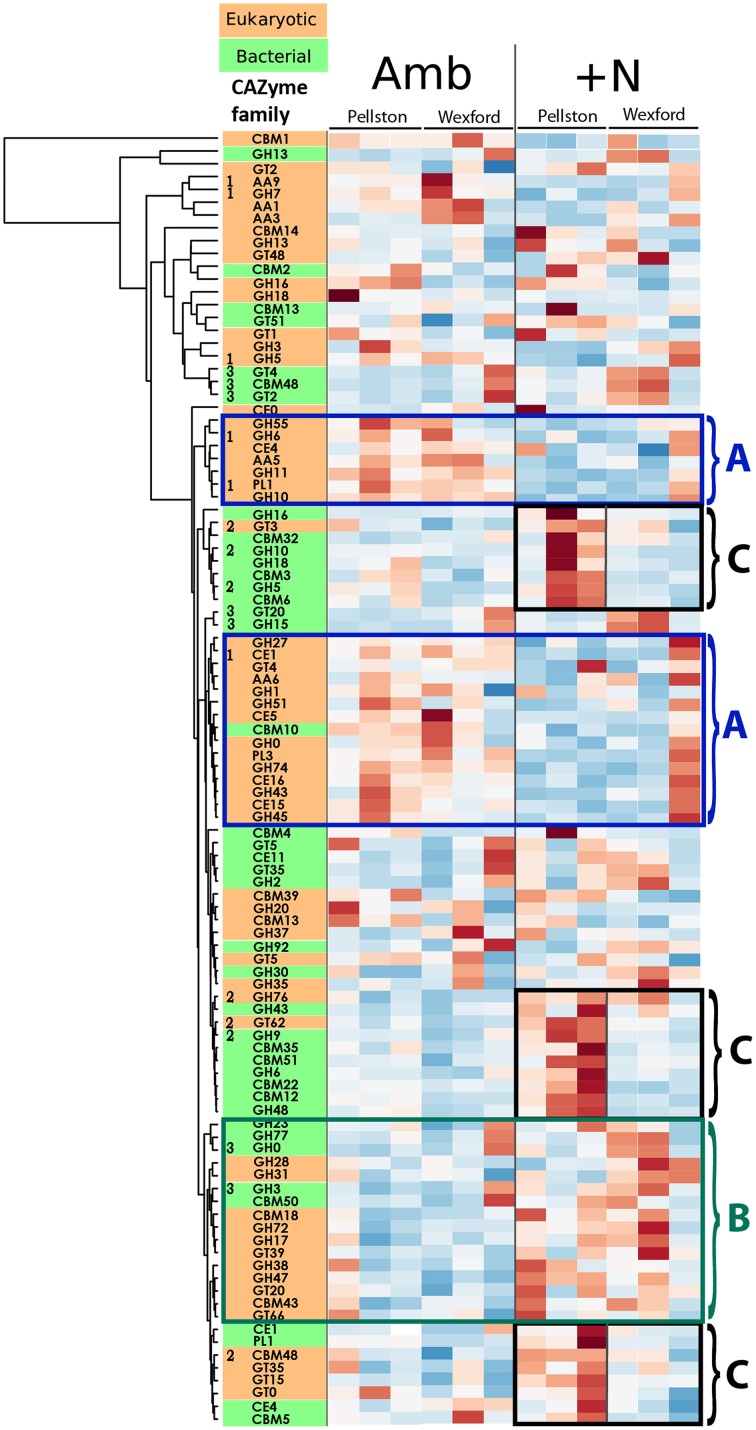
**Heat map of CAZyme transcript abundance by taxonomy**. The 100 most abundant CAZy families identified in forest floor metatranscriptomes (~74 K reads per sample) are presented in the heat map to illustrate trends in the CAZyme abundance datasets. CAZyme families are split into eukaryotic (orange) and bacterial (green) groups based on sequence homology to dbCAN database (Yin et al., 2012). CAZyme family codes: GT, glycosyltransferases; GH, glycoside hydrolases; CE, carbohydrate esterases; PL, polysaccharide lyases; CBM, carbohydrate binding modules; AA, axillary activities (oxidative enzymes). The Y-axis dendrogram clusters the CAZymes by common expression pattern using a two-way hierarchical clustering using the complete linkage method. Data were normalized by row and illustrate relative over-expression (increasing red color) and under-expression (increasing blue color). Results from each of the 12 field samples are shown. Three types of patterns are illustrated by the colored, numbered boxes. Blue boxes labeled “A” illustrate groups of eukaryotic CAZymes (with one exception) that are generally over-expressed in ambient field plots and under-expressed in N-amended field plots, with consistent response at both field sites. The green box labeled “B” illustrates a set of eukaryotic and bacterial CAZymes that become increasingly expressed under N-amended conditions at both field sites. The black boxes labeled “C” illustrate differential responses to N-amendment between the Pellston and Wexford field sites, specifically three groups of CAZymes that are increasingly expressed at Pellston but not at Wexford under N-amendment. Numbers to the left of some CAZyme families are those showing highly significant (*p* < 0.001) ordination vectors in Figure 1. [1, eukaryotic CAZymes enriched in ambient plots in both forests; 2, eukaryotic and bacterial CAZymes enriched in Pellston N-amended plots: 3, bacterial CAZymes enriched in Wexford N-amended plots].

The heat map presented in Figure 2 provides a visual assessment of response trends for the 100 most abundant CAZyme families across the datasets after being divided into kingdom-level groups. These are sorted into rows, defined as clusters of families having similar expression patterns. Eukaryote CAZymes are colored orange and bacterial CAZymes are colored green. Relative CAZyme gene expression is presented as distance from group average for each CAZyme family, with colors ranging from red (over-expressed) to blue (under-expressed). Although not as statistically rigorous as the analysis for Figure 1, the heat map visualization of community gene expression across all of the replicate field samples highlights the ability of this metatranscriptome approach to investigate many bacterial and fungal transcripts simultaneously, and to identify sets of correlated transcripts. By employing a metatranscriptome approach that retains mRNA from both bacteria (non-polyA) and eukaryotes (polyA), we can simultaneously assess the bacterial and fungal constituents of CAZyme families where that particular enzyme activity is present in both. Of the 100 most abundant CAZyme families shown in Figure 2, 15 families contain both bacterial and fungal representatives. The transcript responses to N vary between bacterial and fungal members of those families; in some cases both bacterial and fungal transcripts increase, decrease, or have differential responses to N (data not shown).

Three examples of correlated expression of CAZyme families are illustrated in Figure 2. First, the CAZyme families outlined in blue boxes, labeled “A” show clusters of transcripts that are more abundant under ambient N than in N-amended conditions at both field sites. Of these 22 N-responsive CAZyme families, 21 are eukaryotic (with bacterial CAZyme CBM10 the sole exception) and represent many fungal enzymes with lignocelluloytic activities. The eukaryotic glycosyl hydrolases associated with cellulose degradation, cellobiohydrolase I and II (GH6, GH7), and endoxylanase (GH10), and xylanase (GH11) are all more abundant under ambient N compared to the N-amendment treatment. Second, a set 16 CAZymes, representing both bacteria and eukaryote families (outlined in a green box and labeled B in Figure 2) was enriched in N-amended plots at both of the field sites. The functional roles of many of these CAZymes are varied and a unifying ecological trend is not yet apparent; further study of these families is warranted based on their collective response in geographically separated field sites.

CAZyme families that clustered into the A and B boxes showed similar trends in N-response in both field sites. In contrast, the third example of CAZyme clusters illustrate CAZymes that, under N-amendment, clustered separately by forest location. The Pellston forest site exhibited an increase in expressed CAZymes in both bacterial and fungal families (Figure 2, black boxes labeled C), including a group of CAZyme families that contain multiple bacterial glycoside hydrolases identified as potential cellulases and chitinases (GH9 and GH48), as well as bacterial chitin binding modules (CBM12). Relative to the ambient conditions, the Pellston forest also harbored a higher relative abundance of certain eukaryote CAZymes (examples: GT3, GT10, GT15, GT20, GT35, GT62, GH38, GH47, GH76, and CBM48) under long-term N amendment when compared to the Wexford site.

Taken together, the NMS ordination of all CAZyme families (Figure 1) and the expression heat map of the 100 most abundant CAZyme families (Figure 2) illustrate that the community gene expression profiles in the ambient forest floors are more similar to one another than to their respective N-amended forest floors. This is due primarily to the high relative abundance of multiple eukaryotic CAZymes that are associated with lignocellulose biomass breakdown in forest floor samples under ambient conditions relative to long-term N-amended conditions.

**Figure 3:**
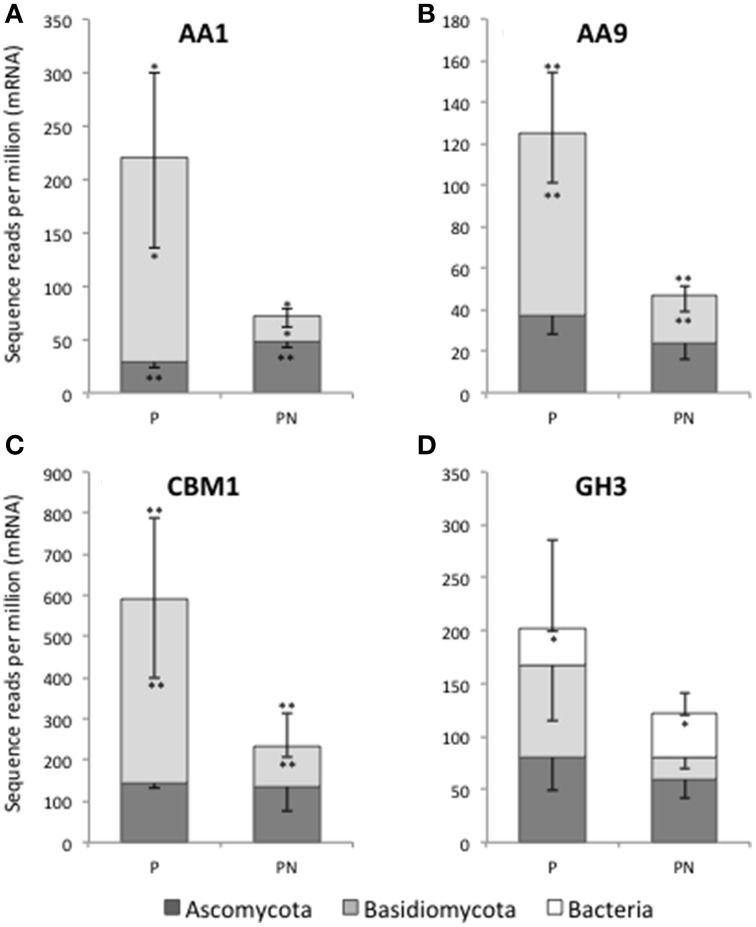
**Fungal phylum-level distribution of select CAZyme transcripts, illustrating differences in expression level between ambient (P) and N-amended (PN) forest floor material at Pellston**. The stacked bar graphs show the relative contribution from Ascomycota (dark gray) and Basidiomycota (light gray) to four CAZyme families in Pellston ambient (P) and N-amended (PN) metatranscriptomes: AA1 (laccase, **A**), AA9 (Cu-dependent lytic polysaccharide monooxygenase, **B**), CBM1 (fungal specific cellulose binding domain, **C**), and GH3 (broad substrate activity including β-D-glucosidases, *N*-acetyl-β-D-glucosaminidases, α-L-arabinofuranosidases, and β-D-xylopyranosidases, **D**). The relative contribution to total GH3 by bacteria is shown in **(D)** (white bars). Bacterial laccase-like enzyme (AA1) was only detected at an average of 1.3 reads per million in Pellston ambient and Pellston +Nitrogen and is not shown in **(A)**. Each column and sub-column represents the average of three replicates and their standard deviation. The upper error bar on each column corresponds to the total number of sequence reads per million mRNA reads. The lower error bars in each sub-column correspond to the standard deviation of the proportion of reads within that taxonomic category (e.g., Ascomycota, Basidiomycota, bacteria). A single ^*^ indicates a significant difference between the ambient and N-amended abundance with a *p*-value of <0.10, based on a pairwise *t*-test. A double ^**^ indicates a significant difference between the ambient and N-amended abundance with a *p*-value of ≤0.05.

Individual CAZyme family abundance profiles were investigated for site and treatment effects; however, the relatively low replication (*n* = 3 field samples per treatment and site) and variable replicate measurements limited our statistical power in this study. As an illustration of how these data can be further explored we compared the abundance of select CAZyme families having well-defined functions at the Pellston field site where the initial PERMANOVA test was significant for nitrogen treatment effects (Figure 3). CAZyme genes that have enough taxonomic representation and sufficient sequence heterogeneity to delineate fungal phylum-level differences were used for further taxonomic analysis. We selected genes involved in lignocellulose and chitin degradation and that had been previously compared by gene-specific approaches in soils at these field sites (Kellner and Vandenbol, 2010). Laccase (CAZyme AA1) and putative Cu-dependent lytic polysaccharide monooxygenase (LPMO; CAZyme AA9) showed a significant reduction in abundance of Basidiomycota transcripts between ambient and N-amended treatments by paired *t*-tests (Figures 3A,B). For laccase, the relative abundance of transcripts associated with Ascomycota increased with N-amendment (Figure 3A). Similar shifts in phylum-level fungal abundance were seen in CAZymes representing the cellulose-binding module (CBM1, Panel C) and the broadly functional GH3 family. These observations further illustrate the overall decrease in fungal transcripts related to lignocellulose biomass degradation under long-term N enrichment that was observed in Figure 2. Furthermore, the reduction in fungal expression is disproportionally associated with a reduction in Basidiomycota gene expression at the Pellston site.

### CAZyme co-expression

**Figure 4:**
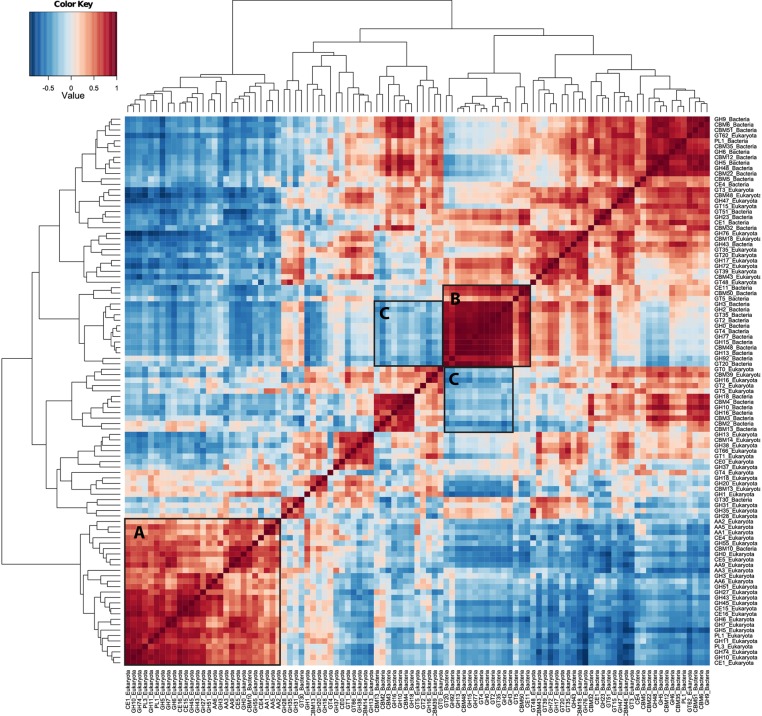
**CAZyme transcript abundance correlation heat map**. The co-expression of many CAZymes in the metatranscriptomes illustrates that this approach is able to detect complex patterns of co-expression among carbohydrate-active genes. Pairwise comparisons of the 100 most abundant CAZyme families that show similar patterns of abundance among the forest floor samples are illustrated as shades of red, while those with differing abundance patterns are shaded blue. Stronger correlations, either positive (red) or negative (blue), are illustrated as darker shades, while pairwise comparisons that show no co-variation are colored white. Hierarchical clustering on X- and Y-axes was generated using complete linkage method. CAZyme abundance correlations were calculated using the Pearson method on Wisconsin double standardized abundance data. Box A indicates a group of eukaryote CAZyme families (with one bacterial family) that are co-expressed. Box B shows a group of bacterial CAZyme families that are also co-expressed, while the C Boxes include bacterial and fungal CAZyme families that are anti-correlated with those in Boxes A and B.

As an exploratory tool in single organism transcriptome studies, the co-expression of genes can be used to identify potential suites of co-regulated or interacting gene suites. Although the interpretation of co-expression patterns in metatranscriptome samples is difficult due to the large number of genes recovered, the transcript correlation technique can still be used to identify suites of correlated, potentially interacting transcripts or organisms. Abundance correlations of the 100 most abundant CAZyme families are shown as a heat map in Figure 4. Hierarchical clustering of these co-expression patterns showed a strong grouping of eukaryote CAZyme families (Figure 4, label A), representing many CAZyme families from all CAZyme classes (GH, GT, PL, CE, AA), most of which contain fungal lignocellulolytic activities. This cluster of genes is also negatively correlated with nearly all of the abundant bacterial CAZyme transcripts. Another striking cluster includes 15 bacterial CAZyme families (Figure 4, label B) that are highly correlated with one another. These families are associated with cleavage of beta- and alpha-galactosidic bonds, glycogen binding, and beta- and alpha-glucosyltransferase activity (GH2, GH3, GH13, GH15). This same cluster is also negatively correlated with both the eukaryotic CAZyme cluster (A), and a cluster of five eukaryotic and six bacterial CAZy families associated with xylan and chitin degradation (GH2, GH5, GH10, GH16; Figure 4, label C), which represent multiple xylan- and chitin-degrading enzymes.

### rDNA and rRNA surveys

#### Relative abundance of fungi and bacteria

Concurrent with the metatranscriptome surveys, we conducted rRNA gene (rDNA) and rRNA qPCR surveys to compare relative abundance between the field sites and N amendment conditions. Across all of the forest floor samples, the amount of extracted DNA or extracted RNA did not differ significantly between the two field sites or between the ambient and N-amended treatments at either site (DNA average (standard deviation), 18 (9) ng/g dry wt, RNA average 0.8 (0.3) ng/g dry wt, *n* = 12). The relative abundance of fungi was highly variable and not significantly different between the two field sites or between the ambient and N-amended treatments at either site when rDNA qPCR was used as the survey tool [rDNA average 9.6 × 10^8^ (5.5 × 10^8^); rRNA average 2.2 × 10^11^ (1.3 × 10^11^)]. In contrast, the relative abundance of bacteria was over three times higher at the Wexford site [3.6 × 10^11^ (6.2 × 10^10^)] when compared to the Pellston site (1.2 × 10^11^ (2.8 × 10^10^*)*; *t*-test *p*-value < 0.001), when rRNA was used. This was not observed when rDNA was used as the template.

#### Fungal and bacterial richness and taxonomic composition

**Figure 5:**
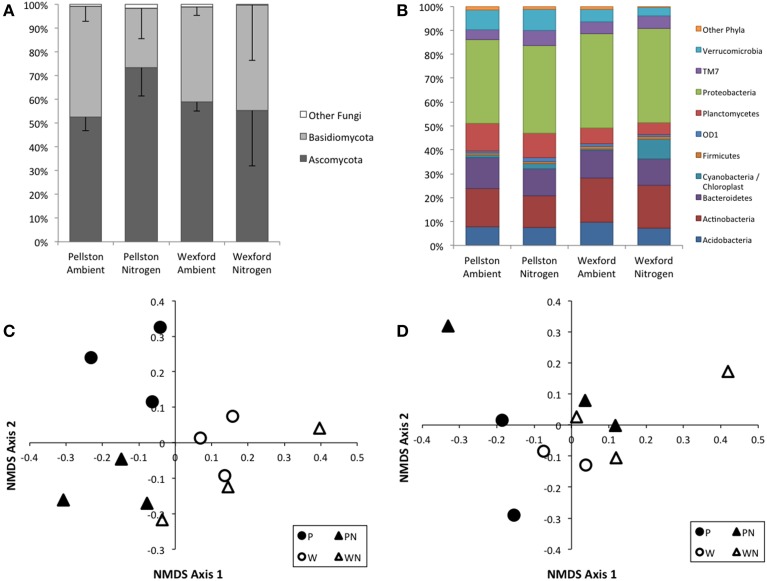
**Fungal and bacterial community composition**. Phylum-level taxonomic distributions from rDNA amplicon sequencing are presented as plot averages (*N* = 3) for fungi **(A)** and bacteria **(B)**. Standard deviations are presented for Ascomycota and Basidiomycota in **(A)**. Standard deviations are not shown in **(B)** for clarity. Nonmetric multidimensional scaling plots illustrating the OTU-level compositional relationships in fungal community **(C)** and bacterial community **(D)** among the two field sites and N-amendment treatments from the rDNA surveys. Each symbol represents a single field plot survey. Fungal community composition showed statistically significant differences by both site and treatment (PERMANOVA, site *p* = 0.001, treatment *p* = 0.015). Bacterial community composition was not significant by both site and treatment (PERMANOVA, site *p* = 0.083, treatment *p* = 0.073).

Richness of fungi or bacteria did not differ significantly between the two field sites or with N-amendment. Ninety-eight to 99% of the fungal OTUs were members of the Basidiomycota or the Ascomycota. The number of Ascomycota in the fungal community increased significantly (*p* = 0.0532) in response to N-amendment at the Pellston site, from an average of 53% to an average of 73% of the community (Figure 5A). A similar shift between the proportion of Ascomycota and Basidiomycota was not observed at the Wexford site. Ordination of the OTU-based composition illustrated the PERMANOVA analysis, where the fungal community composition showed statistically significant differences by both site and treatment (PERMANOVA, site *p* = 0.001, treatment *p* = 0.015; Figure 5C). Ten bacterial phyla comprised over 98% of the 16S rDNA sequences across the sites and N-amendment treatments (Figure 5B). Community composition was not significantly different with site (PERMANOVA, site *p* = 0.083) or N amendment *p* = 0.073) (Figure 5D).

## Discussion

Decomposition of plant biomass in the forest floor is conducted by a diverse group of fungi, other eukaryotes, bacteria and archaea that are highly interactive, and that can respond rapidly and dramatically to changes in environmental conditions such as atmospheric N deposition. The inability to comprehensively and simultaneously survey the total community and their collective functions has severely hampered holistic interpretation of landscape processes, understanding of regional impacts of N-deposition (Janssens et al., 2010; Liu and Greaver, 2010), regional terrestrial carbon cycles, and calculation of C budgets (Friedlingstein et al., 2014).

Our metatranscriptome approach generated a deep gene expression survey (17M data points per sample) of the total decomposer community in maple forest floor material. The approach removed a substantial amount of the rRNA, while retaining both the non-polyA bacterial and the polyA eukaryote mRNA, such that we were able to survey the total community metabolic potential, to compare two maple forests and their responses to N-amendment. Archaeal mRNA was also present in the metatranscriptomes but at a low percent (0.2% of total) and we did not continue to analyze this pool of transcripts. A metaproteomics survey of beech litter similarly demonstrated that the archaea were a low proportion (0–0.2%) of the expressed proteins in the forest floor (Schneider et al., 2012). Using a variety of read-based analyses, we demonstrated the relative representation of bacterial and eukaryotic transcripts in the forest floor metatransriptomes (Table 1, Figure 2), and illustrated good metabolic coverage (KEGG map, Supplementary Figure S1) in this complex microbial community. This approach not only detects specifically targeted expressed genes for which functions are known (Figure 3), but even more importantly it provides a platform for analysis of co-expression among families of bacterial and fungal genes. In the current study we demonstrated this capability using the CAZymes identified in the forest floor metatranscriptomes.

Directly measuring transcriptional activity in microbial communities has been difficult, largely because the high abundance of rRNA often overwhelms mixed-template sequencing. Recent metatranscriptome surveys of soils have used a polyA enrichment to select for eukaryote transcripts and remove rRNA (Bailly et al., 2007; Damon et al., 2012) for survey of soil fungi. Using 454 sequencing without any mRNA enrichment, Urich et al. (2008) and Tveit et al. (2013) applied this approach to farm soils and Arctic peat soils, respectively. Although over 95% of the sequences were rRNA, they demonstrated the feasibility of a comprehensive metatranscriptome approach for soils. Turner et al. (2013) used a similar total RNA 454-based approach to compare the taxonomy of rhizosphere communities among agricultural plants (19 samples having 3 K–144 K small subunit rRNA reads/sample), but analyzed only the rRNA pool. Our approach significantly improves upon these earlier studies by (a) removing the majority of rRNA from an environmental RNA sample without relying on polyA enrichment, and thus (b) retaining the bacterial, archaeal and eukaryote components of a complex community. This approach is now applicable to many studies of interactive metabolism and signaling between eukaryotes and bacteria; for examples, between plants and bacterial pathogens, or among plants, their mycorrhizal fungi and rhizosphere bacteria (Kuske et al., 2015).

We predicted that the complex community in the forest floor material would show responses to N deposition that were manifest in both the bacterial and fungal CAZyme activity profiles, and our results show clearly that both organisms groups are active participants in decomposition and respond to N amended conditions. The bacterial and fungal CAZyme activities are of particular importance in forest floor decomposition processes; they represent the entire process from decomposition of simple sugars to complex polymers such as lignin and chitin. Comparison of the CAZymes in the forest floor metatranscriptomes revealed the relative contributions of fungal and bacterial genes to the pool of expressed genes, and illustrated their different responses to chronic experimental N deposition. We were able to identify about 74 K reads per sample from the metatranscriptomes that mapped to bacterial or fungal CAZymes. Notably, 30 of the 100 most abundant CAZymes were families having good representation of both bacterial and fungal enzymes, which highlights the utility of the non-polyA metatranscriptome approach that could provide simultaneous assessment. While our results clearly show CAZyme gene expression from both fungi and bacteria, a proteomic survey of beech leaf litter identified only fungal enzymes represented in searches for extracellular hydrolytic enzymes (Schneider et al., 2012). This raises interesting questions regarding enzyme abundance and stability in the forest floor environment.

The two natural maple forests used in this study were originally selected based on similarity of floristic and edaphic characteristics. Consistent with this, we found no significant differences between sites in the total extracted nucleic acids or in the relative abundance of fungal biomass (rDNA and rRNA qPCR survey). Forest floor bacterial community taxonomic composition was similar between the two ambient forests; we did not detect significant differences in community richness or OTU composition. The only site difference we detected in the bacterial community was a three-fold higher relative biomass abundance (RT-qPCR) in bacteria at the more southern Wexford forest compared to the Pellston forest. In contrast to the bacterial community, the fungal community OTU composition was significantly different between the two forests when surveyed using the rDNA taxonomic approach, and site was the major constraining variable in a PERMANOVA analysis, explaining 17% of the variability in community composition. Despite the differences in taxonomic composition between the ambient forests, the CAZyme transcript pool in the ambient forests was dominated by similar fungal CAZyme transcripts (Figures 1, 2).

We hypothesized that the metatranscriptomes would illustrate community metabolic shifts that were not necessarily detectable in broader taxonomic surveys based on the bacterial or fungal rDNA. Comparisons of the rDNA and active CAZyme responses showed that the most statistically rigorous responses to N amendment were at Pellston, where the fungal community taxonomic structure and the active CAZyme profiles each differed between ambient and N amended forests. Interesting trends were also noted at Wexford, where a number of new bacterial CAZymes were overexpressed in the Wexford N amended plots (Figure 1) even though bacterial taxonomic differences were small and not significant with 95% confidence.

Despite their floristic and edaphic similarities, the two forests displayed very different responses to 16 years of N deposition. These were readily detected in the profiles of transcribed CAZymes. The Wexford forest transitioned from a fungal-dominated CAZyme profile to a bacterial-dominated profile (Figure 1, quadrant 3). This result demonstrates a large community metabolic change that includes both bacteria and fungi, in response to a subtle but long standing perturbation (chronic N deposition). At the Wexford forest, we did not detect significant differences in overall bacterial taxonomic composition between ambient and N-amended plots, either at the OTU or phylum levels, and it is plausible that the bacterial response to N is due to the naturally abundant bacterial community in forest floor material at this site (as measured by RT-qPCR). Variability among the field replicate samples was high at Wexford, and with only three replicates per N treatment, our statistical power was not high and our CAZyme results must be considered as trends requiring further validation. In contrast to Wexford, the Pellston forest to the north showed statistically rigorous shifts between ambient and N amended plots, transitioning to a mix of bacterial CAZymes and fungal CAZymes that were distinct from the ambient CAZyme profile and from the Wexford forest (Figure 1, quadrant 2).

Environmental and floristic differences between the two forests have been noted in prior studies (e.g., Eisenlord et al., 2013), and these may be driving the different responses of the two forests to N deposition. The northern Pellston forest has marginally cooler temperatures, receiving less rainfall and more ambient atmospheric N than the Wexford forest. The groundcover, seedling recruitment, and understory trees and shrubs also differ substantially between these two forests (Pregitzer et al., 1992; Talhelm et al., 2013), which may change the composition of the forest floor biomass and subsequently the activities of the resident microbial community decomposing that biomass. Indeed, the forest floor litter mass is 50% higher at the southern Wexford site than at the northern Pellston site. The overall response trends of CAZyme activity in maple forest floor communities to N include common features (Figure 2, boxes A, B) and site-specific responses to N (Figure 2, boxes C). Clearly, the responses involve many enzyme activities in both the bacterial and fungal communities, and are influenced by potentially subtle differences in the local geochemistry, weather, and litter characteristics of each forest. More rigorous comparisons from higher field replication would further discern the trends noted here in CAZyme expression patterns.

Despite our observation of no significant difference in measures of fungal biomass or richness in response to N, we noted a common suppression of CAZyme transcription associated with fungal-mediated lignocellulose degradation across N-amended plots at both field sites (Figure 2, boxes labeled A). Our ribosomal survey results indicate that significant phylum-level community changes may help explain the observed CAZyme shifts, primarily the increase in Ascomycota relative to Basidiomycota under N conditions at the Pellston site. This is further supported by the taxonomic distribution of CAZymes contributing to lignocellulose degradation, which show significant reduction in relative abundance at the Pellston forest (Figure 3). Experimental N-amendment has been shown in prior studies to alter the proportion of Ascomycota to Basidiomycota sequences in sequencing surveys (Weber et al., 2013). This observation is consistent with prior fungal community studies at the Pellston site (Hassett et al., 2009; Entwistle et al., 2013) and a microarray-based study at the same Michigan sites, in which Basidiomycota representation of 27 fungal enzymes were diminished, both in richness, and in function, with N-amendment (Eisenlord et al., 2013). In the maple forests surveyed here, Freedman and Zak (2014) showed that under N amended conditions, fungal laccase gene expression and activity declined while activity of bacterial laccase-like multicopper oxidases increased. In the metatranscriptomes the relative abundance of fungal laccase genes (AA1) confirmed the fungal decline in response to N deposition, but the representation of bacterial laccase-like genes was negligible (averaging 1.3 copies per million mRNA reads) in the context of the entire community and did not differ by treatment.

The relative expression of multiple Ascomycota lignocellulose degrading enzymes, including laccase, was not affected experimental N deposition (Figure 3A), indicating the potential for an expanded Ascomycota role in plant biomass degradation under elevated N scenarios. These two broad groups of fungi display a diverse assortment of growth patterns and life styles. Shifts from Basidiomycota fungi to Ascomycota fungi and/or bacterial populations that incompletely decompose plant biomass or do so at slower rates, may account for the observed increases in forest floor biomass and organic matter accumulation in these maple forests under chronic N deposition (Zak et al., 2008). Because fungal and bacterial decomposition processes occur at different rates and to different endpoint products, the differences we have shown in forest floor CAZyme profiles across a relatively small latitudinal gradient have significant implications for modeling efforts aimed at prediction of terrestrial C budgets under concurrent N deposition regimens.

## Conclusions

The metatransriptome approach described here allows one to simultaneously survey the bacterial, archaeal, and fungal mRNA in a complex community, and provides a window into total community metabolism and shifts in response to perturbations. By extracting the CAZymes from the total metatranscriptomes, we were able to demonstrate significant changes in community activities in forest floor material after 16 years of N-amendment, and to observe differences in the ways that two natural forests respond to N-amendment. Higher resolution time course studies with more field replication are needed, and our ability to mine these metatranscriptomes is limited by inability to identify and annotate the entire expressed mRNA collection. Using currently available databases, we were only able to confidently identify about 7% of the transcripts. Clearly a much broader diversity of metabolic capabilities is involved in plant litter decomposition. As genomes of more individual bacteria and fungi are sequenced, and their physiological traits determined by culture-based studies or inferred through genome comparisons, our ability to link elements of these large community datasets to ecologically relevant functions should improve (Kuske et al., 2015). Every new individual genome contains hundreds to thousands of transcribed genes for which no enzyme or regulatory function is yet described, and high-throughput physiological/biochemical studies are sorely needed. For now, it is extremely useful to reliably detect community-level responses to environmental changes in such complex microbial communities, even if one cannot pinpoint a well-characterized biochemical mechanism that underlies the response.

### Conflict of interest statement

The authors declare that the research was conducted in the absence of any commercial or financial relationships that could be construed as a potential conflict of interest.
