# Supplementary material for: Forest floor community metatranscriptomes identify fungal and bacterial responses to N deposition in two maple forests
Source: Front Microbiol. 2015 Apr 23;6:337. doi: 10.3389/fmicb.2015.00337 (PMC4407611; doi:10.3389/fmicb.2015.00337)

Figure S1 – KEGG metabolic map of metatranscriptome data

Global KEGG map with the pathways detected in the total metatranscriptome data, highlighted by relative abundance. Pathway coloring corresponds to relative abundance of transcripts assigned to the pathway with an e-value less than or equal to 1e-5 by UBLAST. Pathways were coded into bins of >1000 reads (red), 100-999 reads (purple), 10-99 reads (blue), and 1-9 reads (black). Pathways with no detected reads were colored light grey.

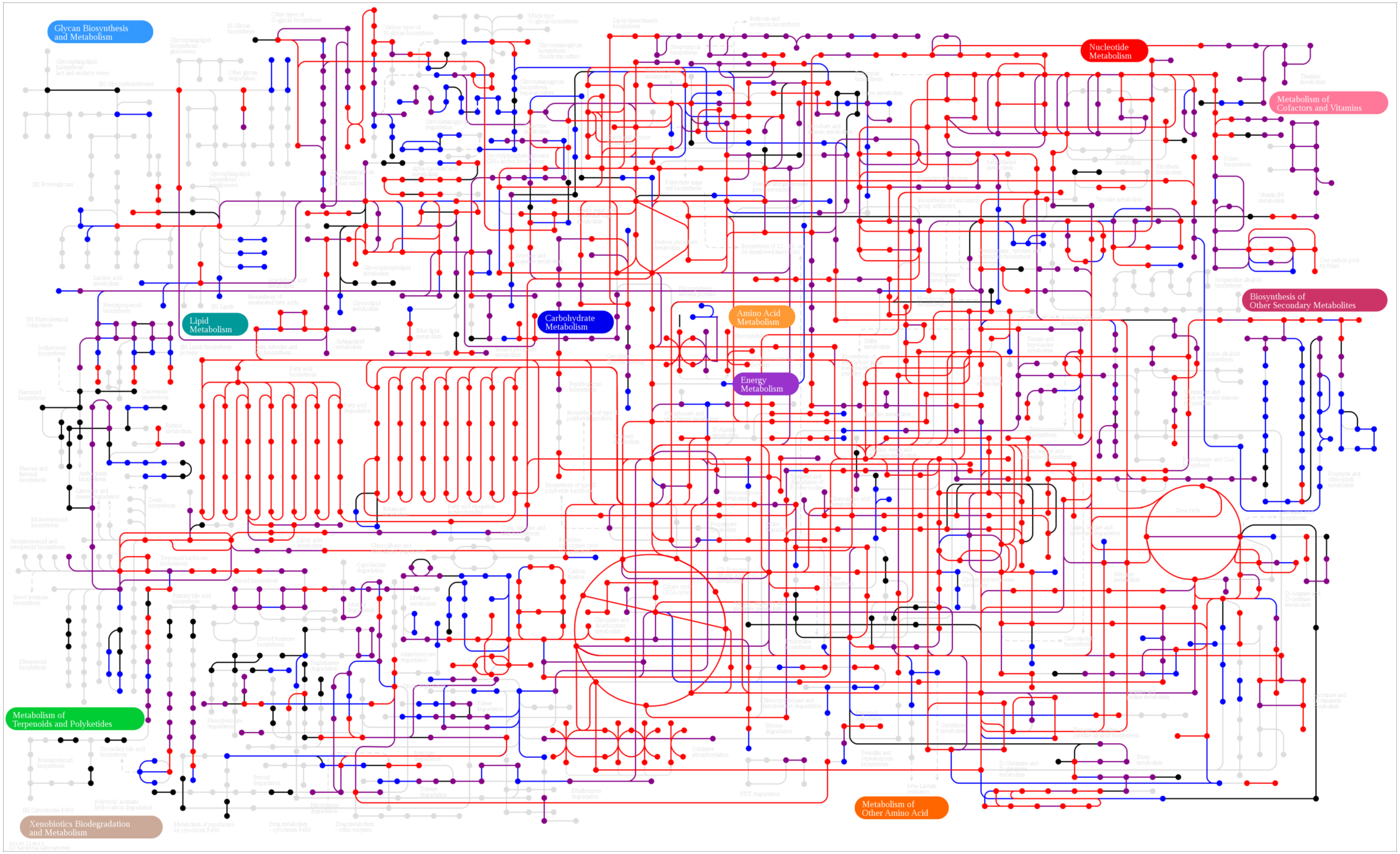

Supplement: Supplementary file 2 [file Image1.PDF]
